# ^18^F-CETO in adrenocortical carcinoma: first clinical-translational study of an adrenocortical-targeting PET tracer

**DOI:** 10.1530/ERC-26-0314

**Published:** 2026-10-05

**Authors:** Adam Edholm, Liang Zhang, James MacFarlane, Tobias Åkerström, Russell Senanayake, Staffan Welin, Agnes Hegedus, Heok K Cheow, Ieva Lase, Peter Stålberg, Azita Monazzam, Daniel Gillett, Luigi Aloj, Per Hellman, Hanna Wargelius, John A Tadross, Gunnar Antoni, Franklin Aigbirhio, Britt Skogseid, Mark Gurnell, Samuel Backman, Anders Sundin, Ruth T Casey, Joakim Crona

**Affiliations:** ^1^ Department of Medical Sciences, Uppsala University, Uppsala, Sweden; ^2^ Department of Endocrinology, Cambridge University Hospitals NHS Foundation Trust, Cambridge, UK; ^3^ Cambridge Endocrine Molecular Imaging Group, University of Cambridge, Cambridge, UK; ^4^ Department of Surgical Sciences, Uppsala University, Uppsala, Sweden; ^5^ Department of Immunology, Genetics and Pathology, Uppsala University, Uppsala, Sweden; ^6^ Department of Nuclear Medicine, Cambridge University Hospitals NHS Foundation Trust, Cambridge, UK; ^7^ Department of Histopathology, Cambridge University Hospitals NHS Foundation Trust, Cambridge, UK; ^8^ Medical Research Council Metabolic Diseases Unit, Institute of Metabolic Science-Metabolic Research Laboratories, University of Cambridge, Cambridge, UK; ^9^ Cambridge Genomics Laboratory, Cambridge University Hospitals NHS Foundation Trust, Cambridge, UK; ^10^ Department of Medicinal Chemistry, Uppsala University, Uppsala, Sweden; ^11^ Wolfson Brain Imaging Centre, University of Cambridge, Cambridge, UK

**Keywords:** adrenocortical carcinoma, positron-emission tomography, translational

## Abstract

The adrenocortical-specific PET tracer [^11^C]metomidate (^11^C-MTO) shows high diagnostic accuracy in adrenocortical carcinoma (ACC), but complex radiochemistry and short half-life limit clinical implementation. The novel tracer para-chloro-2-[^18^F]fluoroethyletomidate (^18^F-CETO) may enable broader use. In this bicentric study, 20 ACC patients underwent PET/CT with ^18^F-CETO and [^18^F]fluoro-2-deoxy-D-glucose (^18^F-FDG), with 235 lesions identified. ^18^F-CETO detected more lesions than ^18^F-FDG at initial staging in 5/13 patients, whereas fewer lesions were detected at re-staging in 5/7 patients (*P* = 0.002). A further 70 patients with ^18^F-CETO or ^11^C-MTO-PET/CT and an anatomical imaging reference were used to explore the utility of adrenocortical imaging. Three adrenocortical imaging phenotypes were defined: homogeneous-high (*n* = 71), homogeneous-low (*n* = 11), and heterogeneous (*n* = 8), which showed differences in overall survival in exploratory analyses. Tumors were characterized by RNA sequencing and immunohistochemistry showing correlation between tracer-uptake to CYP11B1 expression and adrenocortical differentiation. These findings suggest a potential role for ^18^F-CETO in ACC phenotyping and initial staging.

## Introduction

Adrenocortical carcinoma (ACC) is an ultra-rare disease associated with poor prognosis (1). Surgery is recommended as primary treatment, both for newly diagnosed and recurrent disease, when complete tumor removal is feasible (1, 2, 3). Accurate staging and re-staging are, therefore, essential to guide therapeutic decisions. [^18^F]fluoro-2-deoxy-D-glucose (^18^F-FDG) positron emission tomography combined with computed tomography (PET/CT) is considered the gold standard for ACC staging (1, 2, 3). Prior ^18^F-FDG-PET/CT studies report sensitivity and specificity of approximately 95% at both initial diagnosis and detection of recurrence (4, 5, 6, 7). Whether ^18^F-FDG-PET/CT also reflects ACC proliferation, and thereby provides prognostic information, remains debated (8, 9).

In a pan-cancer context, it is the adrenocortical origin that defines ACC and underlies its histopathological diagnosis (10). The functional imaging tracers, [^11^C]metomidate (^11^C-MTO) and [^123^I]iodometomidate (^123^I-IMTO), targeting the adrenocortical enzymes CYP11B1/B2 (11), can be used to stage adrenocortical neoplasias and to phenotype indeterminate lesions *in vivo* (12, 13, 14). While ^123^I-IMTO single-photon emission computed tomography (SPECT) has shown high specificity for ACC diagnosis, the sensitivity was only 38%; whereas ^11^C-MTO-PET/CT demonstrated considerably higher sensitivity (up to 86%) (15). However, CYP11B1/B2-targeted PET tracers have never been directly compared with ^18^F-FDG in ACC. Whether molecular imaging targeting adrenocortical features can distinguish molecular subtypes of ACC, and thus serve to phenotype ACC in a noninvasive manner, remains unknown.

A key limitation of ^11^C-MTO is the short half-life of ^11^C (*t*½ = 20 min), which requires on-site cyclotron production and restricts patient throughput. Para-chloro-2-[^18^F]fluoroethyletomidate (^18^F-CETO), a recently developed PET tracer targeting CYP11B1/B2, benefits from simpler production and a long half-life (^18^F *t*½ = 110 min) compared with ^11^C-MTO-PET/CT and with the potential for superior imaging quality compared with ^123^I-IMTO-SPECT (16, 17). Although early data suggest feasibility, the clinical experience with ^18^F-CETO-PET in ACC remains limited (17). This study was, therefore, designed to evaluate the performance of ^18^F-CETO PET/CT to diagnose and phenotype ACC, both in combination with and compared with ^18^F-FDG PET/CT.

## Materials and methods

### Study design, participants, and ethics

This was a bicentric retrospective cohort study conducted at two tertiary referral centers: Akademiska Sjukhuset (Uppsala, Sweden) and Cambridge University Hospitals NHS Foundation Trust (Cambridge, United Kingdom). ACC patients who had previously undergone ^18^F-CETO-PET/CT and/or ^11^C-MTO-PET/CT between 2010 and 2025 were screened for inclusion. Eligibility criteria were set as follows: i) age ≥ 18 years; ii) histopathological confirmation of ACC; iii) presence of primary and/or metastatic disease at the time of imaging; and iv) availability of data on prior and ongoing treatments. Follow-up data on survival and progression-free survival were collected until September 30, 2025. The analysis plan of this study was drafted at the beginning of the study in 2023, and then finalized before completing data collection. The study was conducted in accordance with the Declaration of Helsinki and was approved by appropriate research ethics committees for Uppsala (EPM Dnrs 2023-00163-01, 2025-00257-02, and 2025-06748-02) and Cambridge (REC: 14/EE/1059). All living patients provided written informed consent.

### Data collection

The Uppsala cohort comprised ACC patients undergoing ^18^F-CETO-PET/CT and/or ^11^C-MTO-PET/CT in routine clinical practice. Between 2023 and 2025, those planned for ^18^F-CETO-PET/CT were additionally considered for enrollment in the prospective PROGRESS-ACC study, which included ^18^F-FDG-PET/CT imaging and collection of frozen tumor biomaterials via surgical resection or core-needle biopsy (FOU2023-00154; registered on August 27, 2023). The Cambridge participants were identified via the service evaluation: ‘An Evaluation of the Management of Adrenocortical Carcinoma at Cambridge University Hospitals NHS Foundation Trust from 2010 to present’ (ID: 5897) and recruited to the ‘Genomic Analysis of Endocrine and Neuroendocrine Tumours’ study. A subset of five Cambridge patients were recruited for a pilot study exploring whole-body ^18^F-CETO-PET/MR. The remainder underwent ^11^C-MTO-PET/CT or ^18^F-CETO-PET/CT as part of standard clinical care. All patients had follow-up imaging every 3–6 months, with a minimum of 5 years of follow-up after radical surgery. Survival data were retrieved through the Swedish and the British population registries.

### Study objectives and outcome measures

The primary objective was to explore the performance of ^18^F-CETO-PET/CT in ACC compared with matched ^18^F-FDG-PET/CT (performed within a 3-month interval). A separate extended cohort was used to determine whether the uptake phenotypes identified in the paired cohort could be reproduced using ^18^F-CETO/11C-MTO-PET/CT, with contrast-enhanced CT serving as the reference. The combined cohorts were used to study exploratory objectives including assessment of the associations between ^18^F-CETO- and ^11^C-MTO-PET/CT uptake patterns with clinical and molecular findings.

The predefined primary outcomes were defined as follows: i) the proportion of ACC lesions characterized as ^18^F-CETO-high/^18^F-FDG-low, ^18^F-CETO-high/^18^F-FDG-high, and ^18^F-CETO-low/^18^F-FDG-high, in patients with matched ^18^F-CETO- and ^18^F-FDG-PET/CT; and ii) the proportion of patients in whom ^18^F-CETO-PET/CT detected more, equal, and fewer lesions than matched ^18^F-FDG-PET/CT. Definitions of high and low uptake are described below.

In the extended cohort, the diagnostic yield of ^18^F-CETO-PET or ^11^C-MTO-PET was compared with that of contrast-enhanced CT. Furthermore, we performed an exploratory evaluation of the clinical-molecular characteristics associated with:

- three different adrenocortical imaging phenotypes: homogeneous-high (all lesions ^18^F-CETO-/^11^C-MTO high), homogeneous-low (all lesions ^18^F-CETO-/^11^C-MTO low), and heterogeneous (at least one ^18^F-CETO-/^11^C-MTO-low uptake tumor and one ^18^F-CETO-/^11^C-MTO-high uptake lesion), and
- tumor ^18^F-CETO-/^11^C-MTO maximum standardized uptake value (SUVmax).

RNA sequencing (Uppsala) and immunohistochemistry (Cambridge) were performed when paired PET scan and tumor tissue were available, defined as ^11^C-MTO-PET/CT or ^18^F-CETO-PET/CT performed ≤3 months prior to tumor tissue acquisition. When available, multiple tumor samples from different tumor sites or time points were included. For unique lesions with more than one tumor sample available, one representative sample was selected.

### PET/CT imaging analysis

Detailed PET/CT imaging protocols are available in the Supplementary materials (see section on Supplementary materials given at the end of the article). Imaging data from PET/CT with ^11^C-MTO-, ^18^F-CETO-, and ^18^F-FDG were initially reviewed in routine clinical practice by nuclear medicine imaging specialists and radiologists. Secondary review was performed at each site (A E and J M) and confirmed by senior nuclear imaging specialists (A S and H C). Tumors were assessed by visual qualitative and semi-quantitative criteria, as detailed below, and dichotomized as having high or low tracer uptake. When no obvious tumor lesions with increased tracer uptake were seen during initial review, contrast-enhanced CT was used to facilitate delineation of suspected tumors for standardized uptake value measurement. Lesion detection was evaluated according to anatomic compartment: lung, bone, liver, peritoneum, lymph nodes (thoracic, retroperitoneal, abdominal), local recurrence, and *in-situ* primary tumor. Lesions were counted per anatomical compartment and capped at 20.

Volumes of interest (VOIs) were manually drawn in three planes to include tumor lesions and avoid adjacent structures, and the voxel with the highest SUVmax was recorded. In scans where no tumor lesions were classified as high uptake, the SUVmax was recorded for the most avid suspected tumor lesion in the scan, using contrast-enhanced CT images to guide the placement of VOIs. The threshold for categorizing lesions as ^11^C-MTO- and ^18^F-CETO-high/-low was the SUVmax greater/less than that of bone marrow. The bone marrow SUVmax was determined by placing a VOI in the L4 vertebral body, avoiding cortical bone. For ^18^F-FDG-PET/CT, the uptake in normal liver was used as the cut-off point to identify ^18^F-FDG-high/-low tumor lesions. Soft tissue lesions measuring less than 5 millimeters on CT were disregarded, as were lesions that were completely necrotic, based on contrast-enhanced CT images.

When evaluating ^18^F-CETO-/^11^C-MTO-PET/CT, without matched ^18^F-FDG-PET/CT scans, CT scans were used for correlation and comparison. Any lesion classified as ‘^18^F-CETO/^11^C-MTO-high uptake’ was considered to represent an ACC tumor. Suspected tumors on CT, classified as low-uptake lesions on PET, were evaluated on serial CT follow-up, with radiological progression considered as confirmation of malignancy. Multidisciplinary team meetings reviewed all cases with low-uptake lesions. When all lesions in a patient, identified on CT, showed high ^18^F-CETO-/^11^C-MTO-PET/CT uptake, PET and CT were considered equivalent. When ^18^F-CETO-/^11^C-MTO-PET identified more lesions than CT, ^18^F-CETO-/^11^C-MTO-PET was classified as superior to CT and vice versa.

### Tumor tissue acquisition

Uppsala: For patients following the prospective PROGRESS-ACC protocol, tumor tissue samples were snap-frozen in liquid nitrogen within 2 h after retrieval, and then preserved at −80°C. When surgery was not planned, trans-abdominal ultrasound-guided core-needle biopsy (18-gauge) was performed, targeting progressive metastatic sites. In addition, any archived frozen tumor tissue samples from the included patients available in Uppsala biobanks at the Endocrine Surgery group or U-CAN (18) biobanks were also retrieved.

Cambridge: Archived formalin-fixed, paraffin-embedded tumor tissue was acquired from the diagnostic histopathology archive to be used for immunohistochemistry analyses. Details on the protocols for tissue and nucleic acid preparation, RNA sequencing, and immunohistochemistry are available in the Supplementary materials.

### Statistical analyses

Statistical analyses were performed with GraphPad Prism (GraphPad Software, version 10.4.2, San Diego, CA, USA) and R (version 4.4.2). Continuous variables were presented as mean ± standard deviation (SD) or median and interquartile range (IQR), as appropriate. For non-parametric data, Spearman’s correlation, Mann–Whitney U, and Kruskal–Wallis tests were used. *P* < 0.05 was considered statistically significant, and all *P*-values were two-tailed. Overall survival (OS) was defined as time from first diagnosis to death or last follow-up. Differences in OS were analyzed with Kaplan–Meier curves, log-rank, and Cox regression tests.

## Results

### Patients

A total of 152 ACC patients undergoing ^18^F-CETO-PET/CT and/or ^11^C-MTO-PET/CT were identified, of whom 90 fulfilled the study criteria and were included for analyses: 30 had ^18^F-CETO-PET/CT and 60 had ^11^C-MTO-PET/CT scans (Supplementary Fig. 1). The patients’ mean age at diagnosis was 55 years and 65% (59/90) were females. Androgen excess was observed in 16% (14/90) of patients, whereas subclinical cortisol excess and clinical evidence of Cushing syndrome were present in 14% (13/90) and 34% (31/90), respectively. At primary diagnosis, 64% (58/90) had locoregional stage (I–III), while 36% (32/90) were primary metastatic (stage IV). The fraction of conventional ACC was 87% (78/90), with 12% (11/90) being the oncocytic subtype and 1% (1/90) being the myxoid subtype. The median Ki-67 index was 18% (IQR: 8–30) (Table 1).

**Table 1:** Patient and disease characteristics of the study cohort. Percentages may not sum to 100 due to rounding. ACC, adrenocortical carcinoma; CETO, para-chloro-2-[^18^F]fluoroethyletomidate; ENS@T, European Network for the Study of Adrenal Tumors; FDG, [^18^F]fluoro-2-deoxy-D-glucose; IQR, interquartile range; SD, standard deviation.

	Study cohort (*n* = 90)	CETO-FDG (*n* = 20)	Extended (*n* = 70)	*P*-value
Age at diagnosis, mean ± SD	54.8 ± 16.3	55.4 ± 17	54.7 ± 16.1	0.86
Male:female, *n*	31:59	4:16	27:43	0.17
Clinical cortisol excess, *n* (%)	31 (34)	4 (20)	27 (39)	0.18
Biochemical signs of cortisol excess without clinical signs, *n* (%)	13 (14)	2 (10)	11 (16)	0.91
Biochemical and/or clinical signs of sex steroid excess, *n* (%)	14 (16)	8 (40)	6 (8)	0.03
ENS@T stage at diagnosis	..	..	..	0.67
I	2	1	1	..
II	31	6	25	..
III	25	5	20	..
IV	32	8	24	..
Ki-67 index, median (IQR)	18 (8–30)	14 (10–25)	20 (7–30)	0.70
Weiss score, median (IQR)	7 (5–7)	7 (5.25–7)	6 (5–7)	0.56
Tumor subtype, *n* (%)	..	..	..	0.14
Conventional ACC, *n* (%)	78 (87)	15 (75)	63 (90)	..
Oncocytic, *n* (%)	11 (12)	5 (25)	6 (9)	..
Myxoid, *n* (%)	1 (1)	0	1 (1)	..
Overall survival in months, median (IQR)	30.5 (11.75–77)	15.5 (6–73.5)	33 (12.75–77.25)	0.08
Mitotane prior to scan, *n* (%)	32 (36)	8 (40)	24 (34)	0.59

### Diagnostic capacity of ^18^F-CETO-PET for staging and re-staging of ACC

Twenty patients had matched ^18^F-CETO- and ^18^F-FDG-PET/CT scans and were included for the primary outcome analysis (Fig. 1A). In these scans, a total of 235 ACC lesions were detected that were classified based on their tracer uptake profile: 21 (9%) lesions were ^18^F-CETO-high/^18^F-FDG-low, 162 (69%) were ^18^F-CETO-high/^18^F-FDG-high, while 52 (22%) were ^18^F-CETO-low/^18^F-FDG-high. There were no ^18^F-CETO-low/^18^F-FDG-low lesions (Fig. 1B, C, D).

**Figure 1:**
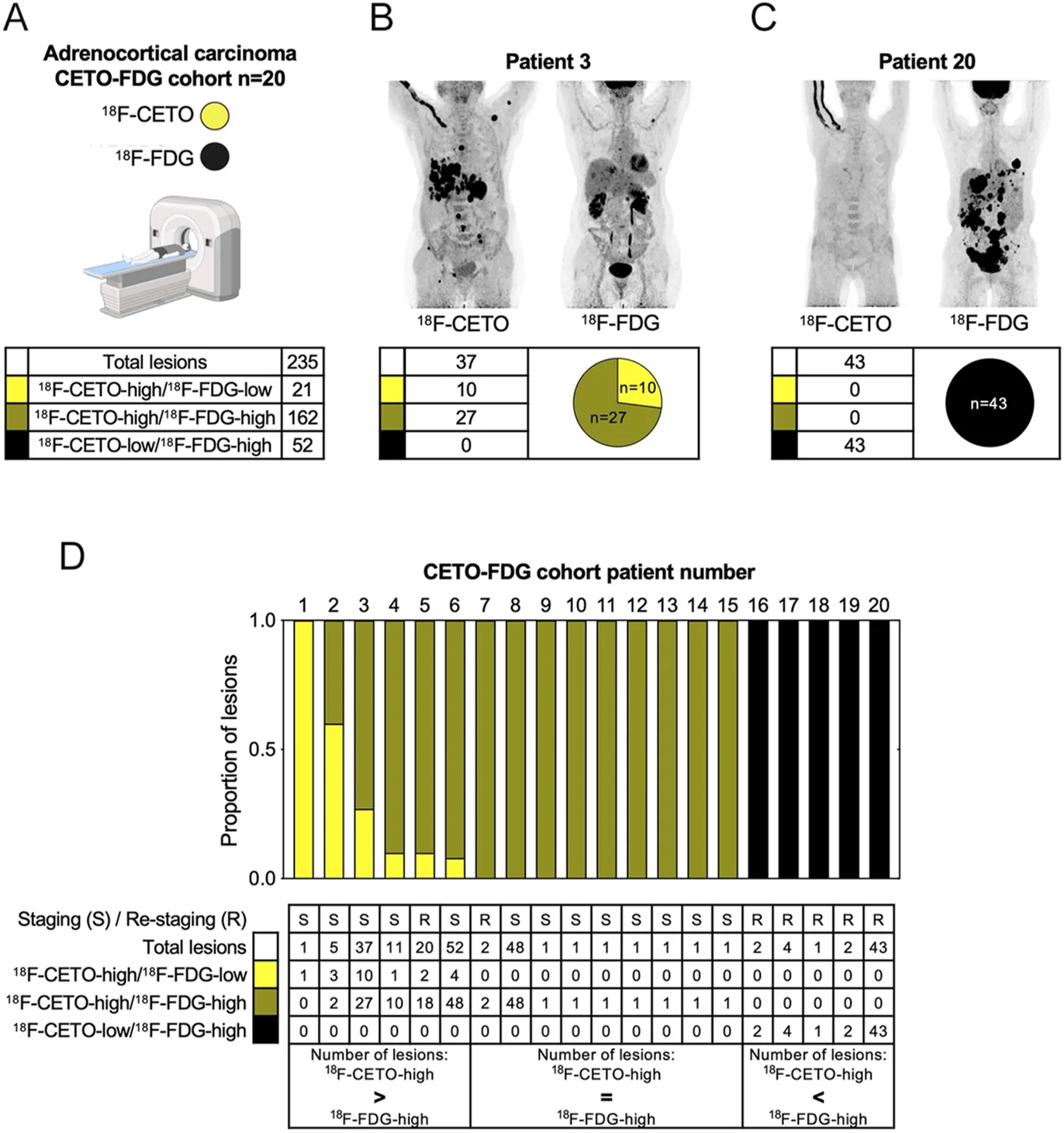
(A, B, C, D) CETO-FDG cohort and primary outcome. (A) The ^18^F-CETO and ^18^F-FDG cohort included 20 adrenocortical carcinoma patients for primary outcome analysis. (B and C) Representative 3D maximum intensity projection PET image exemplifying the methodology to classify lesions as ^18^F-CETO-high/^18^F-FDG-low (yellow), ^18^F-CETO-high/^18^F-FDG-high (dark yellow), and ^18^F-CETO-low/^18^F-FDG-high (black). (D) Primary outcome: proportion of ^18^F-CETO-high/low ACC lesions detected on PET/CT as compared with matched ^18^F-FDG-PET/CT. Part of this figure was made using BioRender.com. ^18^F-CETO, para-chloro-2[^18^F]fluoroethyletomidate; ^18^F-FDG, [^18^F]fluoro-2-deoxy-D-glucose; R, re-staging; S, staging.

On a per-patient basis, imaging results differed according to the clinical imaging setting. At primary staging, ^18^F-CETO detected more lesions than ^18^F-FDG in 5 of 13 patients (38%) and an equal number of lesions in the remaining eight patients (62%). By contrast, at disease re-staging (*n* = 7), ^18^F-CETO detected more lesions in one patient (14%) and an equal number of lesions in one patient (14%), whereas fewer lesions were detected in five patients (71%) compared with ^18^F-FDG (*P* = 0.002). All five patients in whom ^18^F-CETO detected fewer lesions than ^18^F-FDG had ongoing or prior mitotane therapy.

### Adrenocortical specific PET imaging in staging and re-staging of ACC

To further explore tracer uptake patterns observed in the CETO-FDG cohort, we analyzed 70 additional patients, including 10 imaged with ^18^F-CETO-PET/CT and 60 with ^11^C-MTO-PET/CT, who showed broadly comparable disease characteristics (Table 1), with the exception of a higher frequency of biochemical and/or clinical sex steroid excess (40 vs 8%, *P* = 0.03) in the CETO-FDG cohort. Clinical cortisol excess was less frequent in the CETO-FDG cohort (20 vs 39%), although this difference was not statistically significant (*P* = 0.18). In patients who underwent ^18^F-CETO-PET/CT (*n* = 10), there were 50 ACC lesions detected in total, with 32 classified as ^18^F-CETO-high and 18 as ^18^F-CETO-low. ^11^C-MTO-PET/CT (*n* = 60) detected a total of 523 lesions, of which 486 were ^11^C-MTO-high and 37 were ^11^C-MTO-low.

At primary staging of ACC, ^18^F-CETO PET detected an equal number of lesions compared with CT in two of three patients (66%) and fewer lesions in one patient (33%). ^11^C-MTO PET detected more lesions in 3 of 29 patients (10%), an equal number in 21 of 29 patients (73%), and fewer lesions in 5 of 29 patients (17%) compared with CT (*P* = 0.17).

By contrast, at disease re-staging, ^18^F-CETO PET detected more lesions than CT in 1 of 7 patients (14%), an equal number of lesions in 2 of 7 patients (29%), and fewer lesions in 4 of 7 patients (57%). ^11^C-MTO PET detected more lesions in 15 of 31 patients (48%), an equal number in 12 of 31 patients (39%), and fewer lesions in 4 of 31 patients (13%) compared with CT (*P* = 0.01). Nine lesions were considered completely necrotic and excluded from analysis.

In addition, we identified a distinct adrenocortical imaging pattern characterized by heterogeneous tracer uptake, which was not observed in the CETO-FDG comparison cohort (Fig. 2A, Supplementary Figs 2 and 3). One patient undergoing ^18^F-CETO-PET/CT and seven patients who had ^11^C-MTO-PET/CT exhibited heterogeneous scans with both high- and low-uptake lesions. In these 8 patients, there were 83 lesions identified on contrast-enhanced CT, of which 24% (20/83) were classified as low-uptake. Thus, our analyses of the CETO-FDG and the extended cohorts defined three adrenocortical imaging phenotypes: homogeneous-high, homogeneous-low, and heterogeneous that were used for further explorative analyses.

**Figure 2:**
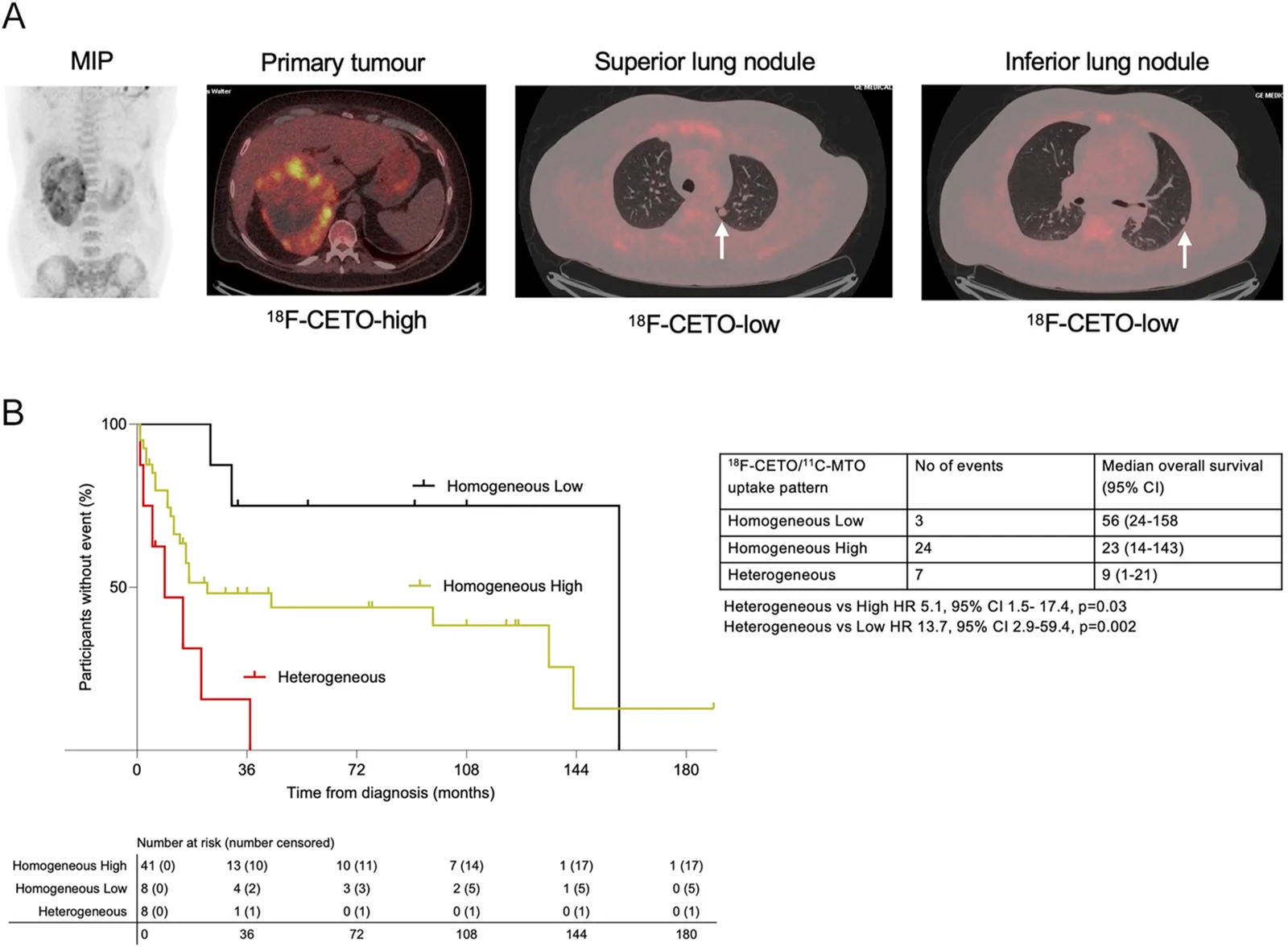
(A and B) Survival in adrenocortical carcinoma with different uptake patterns. (A) Representative adrenocortical carcinoma patient with heterogeneity, demonstrating high ^18^F-CETO uptake in the primary tumor and low ^18^F-CETO uptake in synchronous lung metastases. (B) Kaplan–Meier curves comparing overall survival of adrenocortical carcinoma with ENS@T stage III and IV at primary diagnosis. Patients were stratified by uptake patterns. 11C-MTO, [^11^C]metomidate; ^18^F-CETO, para-chloro-2 [^18^F]fluoroethyletomidate; CI, confidence interval; ENS@T, European Network for the Study of Adrenal Tumours; HR, hazard ratio.

### Clinico-pathological correlates of ^18^F-CETO/^11^C-MTO tumor uptake patterns

Based on the hypothesis that ^18^F-CETO and ^11^C-MTO tumor uptake patterns reflected disease biology with potential prognostic relevance, the full cohort (*n* = 90) was stratified into three groups (Table 2): 79% (71/90) with homogeneous-high uptake, 9% (8/90) with heterogeneous uptake, and 12% (11/90) with homogeneous-low uptake. The heterogeneous group showed more aggressive disease characteristics: median Ki-67 was 30% compared with 14.5 and 18% for the homogeneous-high and homogeneous-low groups, respectively (*P* = 0.01). Similarly, the heterogeneous group had a median Weiss score of 8, versus 6 and 7 for the homogeneous-high and -low groups, respectively (*P* = 0.01). The oncocytic subtype of ACC, generally recognized as being less aggressive than conventional ACC, accounted for 36% (4/11) of the homogeneous-low uptake group, but merely 10% (7/71) in the homogeneous-high group, and was not observed in the heterogeneous group (*P* = 0.04).

**Table 2:** Clinical characteristics in adrenocortical carcinoma imaging subtypes. Percentages may not sum to 100 due to rounding. ACC, adrenocortical carcinoma; CETO, para-chloro-2-[^18^F]fluoroethyletomidate; ENS@T, European Network for the Study of Adrenal Tumors; MTO, [11C]metomidate; IQR, interquartile range; SD, standard deviation.

	MTO/CETO-high (*n* = 71)	Heterogeneous MTO/CETO (*n* = 8)	MTO/CETO-low (*n* = 11)	*P*-value
Age of patient at diagnosis, mean ± SD	54.7 ± 15.9	56.34 ± 16.8	54.21 ± 15.8	0.89
Male:female, *n*	24:47	4:4	3:8	0.61
Clinical cortisol excess, *n* (%)	25 (35)	5 (63)	2 (18)	0.16
Biochemical signs of cortisol excess without clinical signs, *n* (%)	12 (17)	1 (13)	0	0.41
Biochemical or clinical signs of sex steroid excess, *n* (%)	11 (15)	1 (13)	2 (18)	0.99
ENS@T stage at diagnosis, *n*	..	..	..	0.07
I	1	0	1	..
II	27	0	3	..
III	21	2	2	..
IV	21	6	5	..
Ki-67 index, median (IQR)	14.5 (7–30)	30 (25–56.75)	18 (14.5–24.5)	0.01
Weiss score, median (IQR)	6 (4.25–7)	8 (6–8)	7 (6–7.5)	0.01
Tumor subtype, *n* (%)	..	..	..	0.04
ACC, *n* (%)	63 (89)	8 (100)	7 (64)	..
Oncocytic, *n* (%)	7 (10)	0	4 (36)	..
Myxoid, *n* (%)	1 (1)	0	0	..
Overall survival in months, median (IQR)	29 (12–77)	7.5 (2.75–19.5)	56 (31–108)	0.004
Mitotane prior to scan, *n* (%)	23 (32)	0	9 (82)	0.002

As previously highlighted, the pattern of adrenocortical tracer uptake depended on the clinical scenario: all eleven scans showing homogeneous-low tumor uptake were performed at re-staging while 6 of 8 scans with heterogeneous tumor uptake were performed at primary staging. Previous mitotane exposure (defined as reaching therapeutic concentration and/or 6 months duration of treatment prior to imaging) was strongly associated with homogeneously low tracer uptake: 81% (9/11) of homogeneous-low uptake scans were performed in patients who had previous or ongoing mitotane. In comparison, 32% (23/71) in the high-uptake group and none in the heterogeneous group (*P* = 0.002) had a history of previous mitotane exposure.

To evaluate the prognostic impact of the adrenocortical-imaging patterns, we excluded patients with ENS@T stages I and II at primary diagnosis, in order to reduce heterogeneity (Supplementary Table 1). The median overall survival from primary diagnosis was lower in the heterogeneous group (9 months) compared with the homogeneous-high (23 months; hazard ratio (HR): 5.06, 95% confidence interval (CI): 1.47–17.43; *P* = 0.029, Fig. 2B) and homogeneous-low groups (56 months; HR: 13.7, 95% CI: 2.92–59.39; *P* = 0.002, Fig. 2B).

### ^18^F-CETO imaging findings and CYP11B1 expression are associated with mitotane treatment

We further explored correlates of ^18^F-CETO and ^11^C-MTO SUVmax among the clinico-pathological variables with no difference observed based on the hormonal status of the disease. Instead, the most powerful determinant of ^18^F-CETO SUVmax was mitotane, with lower uptake in 14 patients with prior or ongoing mitotane treatment (median 3.7, IQR 2.4–14.4) compared with 16 untreated patients (median: 24.7; IQR: 11.4–40.1; *P* = 0.005). There was no difference in ^11^C-MTO SUVmax among 35 mitotane-treated (median: 22.1; IQR: 12.5–34) and 25 therapy-naïve patients (median: 28.3; IQR: 17.2–37.1; *P* = 0.41). To exclude unspecific ^18^F-CETO-tracer binding and/or pharmacokinetic interference related to mitotane treatment, we generated RNA-sequencing data from 20 patients with 26 unique ACC lesions having matched ^18^F-CETO- (*n* = 13) or ^11^C-MTO-PET (*n* = 13, Fig. 3A, Supplementary Fig. 4). CYP11B1 was significantly upregulated in CETO/MTO-high lesions (Fig. 3B, Supplementary Fig. 4), and there was a strong correlation between *CYP11B1* RNA expression and ^18^F-CETO SUVmax (*R* = 0.85, *P* = 0.0005, Fig. 3C and D, Supplementary Fig. 4). The correlation slope was less pronounced for *CYP11B1* RNA expression and ^11^C-MTO SUVmax (*R* = 0.61, *P* = 0.03). Only a moderately positive linear relationship (*R* = 0.4, *P* = 0.16, Supplementary Fig. 4) between the semi-quantitative CYP11B1 immunostaining score and SUVmax was seen. Two metastases with negative CETO uptake were resected and demonstrated absence of CYP11B1 immunostaining, with both patients having a history of mitotane treatment.

**Figure 3:**
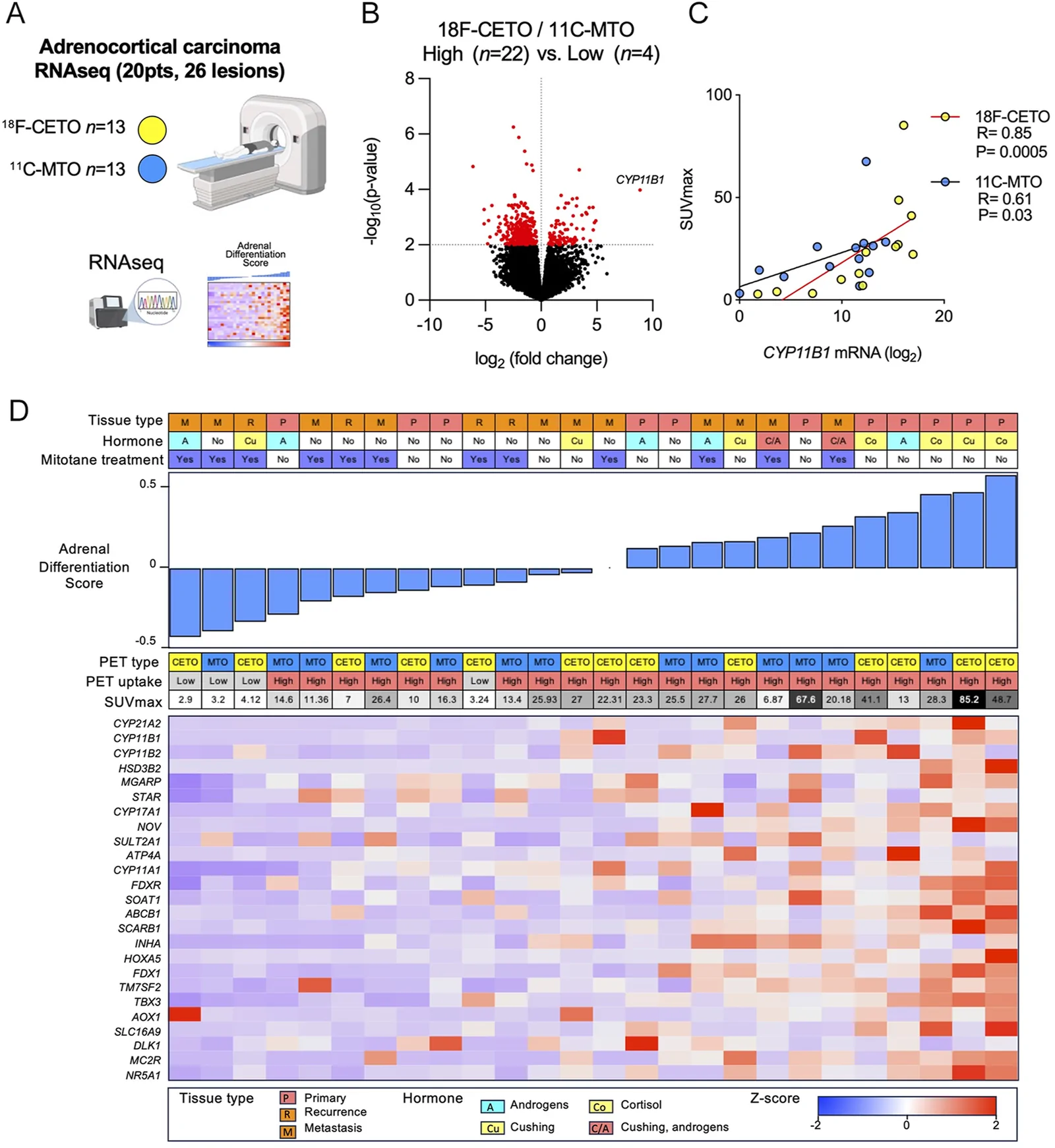
(A, B, C, D) RNA sequencing and immunohistochemistry results. (A) The RNAseq cohort comprising 20 patients with 26 tumor samples and paired ^11^C-MTO (blue) and ^18^F-CETO (yellow) scans. (B) Volcano plot with differentially expressed RNA transcripts between ^18^F-CETO/^11^C-MTO-high and -low groups. (C) Spearman correlation between log2-normalized CYP11B1/B2 gene expression and ^18^F-CETO-PET (yellow)/^11^C-MTO-PET (blue) SUVmax. (D) Correlation heatmap of variables including ^18^F-CETO-/^11^C-MTO-PET SUVmax, CYP11B1/B2 gene expression, and ADS. Part of this figure was made using BioRender.com. 11C-MTO, [^11^C]metomidate; ^18^F-CETO, para-chloro-2 [^18^F]fluoroethyletomidate; A, androgen; ADS, adrenocortical differentiation score; C/A, Cushing and androgens; Co, cortisol; Cu, Cushing; M, metastasis; P, primary tumor; R, recurrence; SUVmax, maximum standardized uptake value.

### ^18^F-CETO-PET tumor uptake reflects adrenocortical differentiation in ACC

We hypothesized that the ^18^F-CETO-low phenotype reflected not only low CYP11B1 expression but also mitotane-associated adrenocortical dedifferentiation. We inferred the adrenocortical differentiation score for each lesion and demonstrate linear correlation with ^18^F-CETO- (*R* = 0.86, *P* = 0.0003) and ^11^C-MTO-PET (*R* = 0.58, *P* = 0.04, Supplementary Fig. 5). To further explore the potential of functional imaging-based phenotyping we inferred the specific adrenocortical zonation scores with ^18^F-CETO SUVmax correlating with zona fasciculata (*R* = 0.85, *P* = 0.0006) and zona reticularis differentiation (*R* = 0.61, *P* = 0.03). Although ^18^F-FDG SUVmax did not correlate with Ki-67 at the cohort level (*n* = 20, *P* = 0.11), selected case examples illustrate how combined ^18^F-CETO and ^18^F-FDG PET provides complementary biological information, enabling *in vivo* discrimination of distinct ACC phenotypic patterns (Fig. 4).

**Figure 4:**
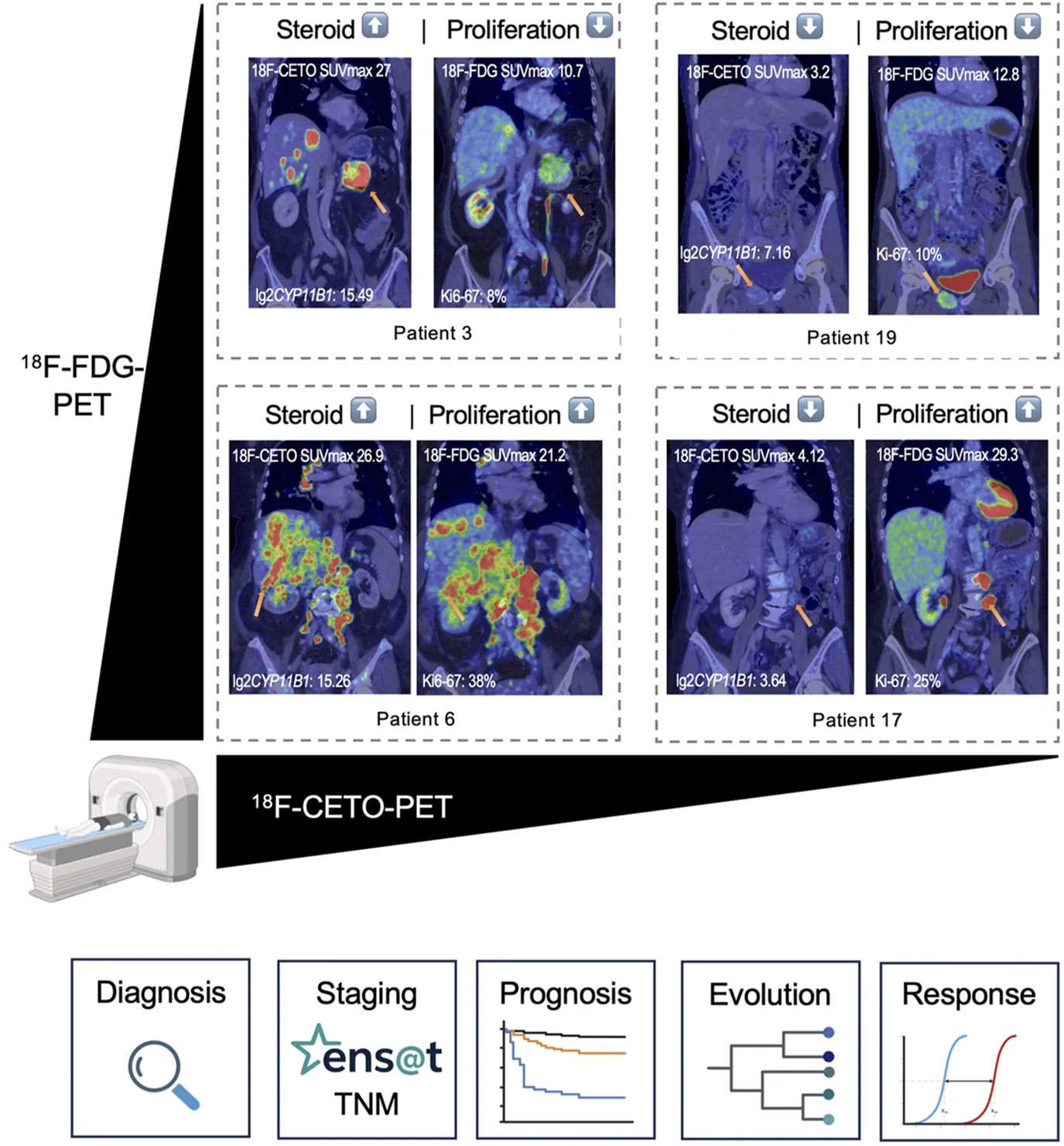
Vision for future use of combined ^18^F-CETO and ^18^F-FDG imaging in adrenocortical carcinoma. Four representative cases from the CETO-FDG cohort with tumor SUVmax measured in ^18^F-CETO- and ^18^F-FDG-PET scans and the corresponding log2-normalized CYP11B1 gene expression and Ki-67 index. The red arrows indicate the adrenocortical carcinoma tumor that was used for tumor sample acquisition and SUVmax measurements. An overview of future potential clinical and research applications is shown below. Part of this figure was made using BioRender.com. ^18^F-CETO, para-chloro-2[^18^F]fluoroethyletomidate; ^18^F-FDG, [^18^F]fluoro-2-deoxy-D-glucose; SUVmax, maximum standardized uptake value.

## Discussion

This is the first study to evaluate ^18^F-CETO-PET in ACC. With 90 patients, it constitutes the largest cohort to date assessing adrenocortical-specific PET tracers, demonstrating that ^18^F-CETO is a highly promising tool for the primary staging of therapy-naïve disease. Beyond staging, the integration of ^18^F-CETO and ^18^F-FDG PET provides a novel platform for noninvasive phenotyping, offering high-resolution insights into the spatiotemporal evolution of tumor biology and its response to treatment.

^11^C-MTO-PET has been in clinical use for over 20 years, and most prior work has focused on its adrenocortical specificity, its use in characterization of adrenal lesions, and its utility in primary aldosteronism (15, 19). Although some cohorts included patients with ACC and demonstrated high sensitivity and specificity in non-necrotic tumors (13, 15, 20), sample sizes have been too limited to properly evaluate its performance for disease characterization. Prior studies suggest that ACC generally exhibits higher tracer uptake than benign lesions (13) and that mitotane treatment reduces uptake (21). Collectively, these studies support the high specificity of ^11^C-MTO for adrenocortical tissue and its utility in diagnostic and primary staging settings. In our cohort, ^18^F-CETO demonstrated performance comparable to ^11^C-MTO for the primary staging of ACC: no untreated primary tumor in our cohort was classified as low uptake, and the proportion of untreated metastatic lesions with low uptake was comparable to ^11^C-MTO. In addition, the present results generate the hypothesis that PET/CT with ^18^F-CETO may have a higher lesion detection rate at primary staging than with the current gold standard ^18^F-FDG. This suggests potential utility of ^18^F-CETO-PET both for distinguishing ACC from other malignant lesions and as a potential new ‘gold standard’ for primary staging. A critical question is whether the higher lesion detection rate of ^18^F-CETO over ^18^F-FDG translates into clinical utility. In our cohort, the identification of additional CETO-positive lesions was concentrated in a few patients and did not change immediate treatment strategies, primarily because these patients already harbored advanced systemic disease. However, high-sensitivity adrenocortical imaging is particularly decisive in the preoperative setting. At initial staging, uncovering even a single previously undetected distant metastasis could prevent non-beneficial adrenalectomies and prompt early systemic therapy.

Our cohort was relatively large and designed to explore the use of ^18^F-CETO, together with ^18^F-FDG, in multiple clinical settings for *in vivo* characterization of ACC. The 20 patients with matched ^18^F-CETO- and FDG-PET/CT showed significant differences in tracer uptake patterns that reflected CYP11B1 expression, adrenocortical differentiation, and tumor proliferation rate. This opens the possibility to study these two hallmarks of ACC *in vivo* with unprecedented spatial resolution, and we propose three different adrenocortical imaging phenotypes in ACC. The most commonly observed was the homogeneous-high pattern that we believe to be representative of ACC that has not undergone any major shift in tumor biology. This contrasts with the homogeneous-low group that was only observed at disease re-staging, when a majority of patients had previously received mitotane, a drug known to affect the expression of adrenal steroidogenic enzymes (22). The two low-uptake scans performed in patients classified as non-mitotane-treated were both cases of late recurrence, and one of these patients had received a short course of adjuvant mitotane (∼1 month). However, demonstrating a causal relationship between mitotane exposure and this imaging phenotype is beyond the scope of this study. In this regard, interesting questions to address in future research are why this imaging phenotype only develops in a subgroup of patients (nine out of 32 mitotane-treated patients in this study), and whether this reflects therapy response or possible therapy resistance.

The third adrenocortical imaging phenotype, the heterogeneous uptake pattern, was detected in treatment-naïve stage III–IV patients and appears to be a harbinger of aggressive biology. This suggests spontaneous tumor evolution with acquisition of more aggressive characteristics and reduced adrenocortical differentiation. These patients showed a significantly higher Ki-67 index and poor prognosis in terms of OS. Unfortunately, none of these low-uptake lesions in patients with heterogeneous scans were sampled for analyses, and a distinct biological phenotype cannot be further evaluated by our study. An intriguing possibility would be a potential correlation between steroid profiling and tracer uptake phenotype. While many patients in this study had undergone steroid profiling, it was rarely synchronized with PET imaging and, therefore, difficult to correlate with tracer uptake.

### Limitations

The cohort described in this study was heterogeneous, representing the entire clinical spectrum of ACC, from untreated primary tumors *in situ* to heavily pre-treated metastatic disease. While this design is conducive to hypothesis generation and exploration of tracer potential, it is not a design suitable to confirm correlations, or to determine imaging sensitivity and specificity, in various clinical scenarios. The timing of PET imaging relative to treatment represents an important limitation of this retrospective study. Consequently, the observed differences in survival between uptake groups may partly reflect differences in treatment exposure and timing of imaging rather than intrinsic tumor biology. Restricting the analysis to treatment-naïve patients could reduce this confounding but would introduce selection bias by excluding patients with longer survival imaged later in their disease course. We, therefore, consider the observed association between tracer uptake and survival exploratory and hypothesis-generating. We initially intended to analyze progression-free survival; however, given the heterogeneity of the cohort and the variable timing of PET imaging relative to treatment, we considered the risk of bias too substantial to allow for a robust analysis and, therefore, excluded it. In addition, the cohort reflects patients at two tertiary centers and may, therefore, not be fully representative of an unselected ACC population. In particular, frail or elderly patients with advanced disease may be underrepresented, as they are less likely to be referred for specialized imaging and treatment.

A methodological limitation of our study concerns the definition and classification of lesions. In the absence of a single reference standard capable of reliably classifying all lesions, the use of a composite reference standard is an established approach in diagnostic accuracy studies (23, 24, 25). In our study, histopathological verification of every individual lesion was neither clinically nor ethically feasible; therefore, we used a composite clinical and imaging reference standard incorporating histopathology where available, together with FDG uptake, anatomical progression on CT, and clinical follow-up. Although this approach reflects real-world clinical assessment, incorporation of CETO/MTO uptake into the reference standard introduces a potential risk of incorporation bias, which could theoretically overestimate the diagnostic performance of the novel tracer.

To mitigate this, lesions demonstrating high FDG uptake or clear anatomical progression on CT were classified as ACC lesions regardless of CETO/MTO status. Furthermore, because CETO/MTO targets specific adrenocortical enzymes, high tracer uptake provides high biological specificity for tissue of adrenocortical origin.

A related limitation concerns the intentionally conservative definition of high tracer uptake, which was chosen to provide a consistent and clear threshold for phenotyping across the cohort. The low-uptake tumors were rarely completely photopenic, and an experienced nuclear medicine specialist would likely have identified at least some of these lesions, particularly with the aid of CT. Nevertheless, regardless of the exact threshold applied, lesions with low CETO/MTO uptake represent a diagnostic challenge. This is illustrated by two patients in whom recurrent low-uptake lesions were initially overlooked and only identified upon subsequent review. Future prospective studies using independent clinical follow-up or blinded panel adjudication, together with standardized image interpretation criteria, are warranted to establish the true diagnostic performance of CETO/MTO without reliance on index-tracer findings.

The tentative identification of a heterogeneous uptake pattern, with possible prognostic significance, is tempered by the small number of patients, the lack of biomaterials, and the lack of matched contemporaneous ^18^F-FDG-PET imaging in this group of patients. Confirmation of this pattern and its potential prognostic impact awaits future studies. Finally, the lack of concurrent ^18^F-CETO/^11^C-MTO-PET-CT imaging in this cohort limits our ability to directly compare the performance of these tracers against each other.

### Future directions

To validate and to further explore the findings of this study, we suggest a large, prospective, multi-center study using longitudinal ^18^F-CETO- and ^18^F-FDG-PET. Patients may be included during the primary diagnostic workup and then subjected to long-term follow-up, optimally with repeated PET imaging at recurrence/progression. This design would enable confirmation of the clinical utility of ^18^F-CETO alone and in combination with ^18^F-FDG-PET for the initial characterization of intermediate potentially malignant adrenal lesions, to assess the sensitivity and specificity for primary tumor staging as well as for re-staging of recurrent and progressive disease. In particular, heterogeneous-uptake at diagnosis and a homogeneous uptake at recurrence/progression should be further investigated, with a focus on underlying mechanisms and clinical utility. Such a study should also be appropriately integrated with collection and characterization of biomaterials. Assuming a very high sensitivity of ^18^F-CETO-PET in this setting, such a study would likely need to include 80–100 indeterminate intermediate potentially malignant adrenal lesions subjected to several years of follow-up (12), an undertaking that would require international collaboration and resources from multiple centers.

## Conclusion

Our findings support a potential role for ^18^F-CETO-PET/CT in the characterization and staging of ACC. The complementary information provided by combined ^18^F-CETO- and ^18^F-FDG-PET/CT may facilitate biologically informed phenotyping of ACC and improve assessment of tumor heterogeneity, individualized management, and treatment response (Fig. 4). Further prospective international studies with longitudinal imaging and molecular profiling are warranted to determine the clinical utility of this approach.

## Supplementary materials



## Declaration of interest

The authors declare that there is no conflict of interest that could be perceived as prejudicing the impartiality of the work reported.

## Funding

The research position of JC is funded by Cancerfonden, and LZ was funded by China Scholarship Council No. 202106370081. JM, LA, MG, and RC are supported by the Cambridge NIHR Biomedical Research Centre (NIHR203312). This work was supported by grants to JC from Cancerfonden, Svenska Läkaresällskapet, Lions Cancerforskningsfond Uppsala, Åke Wibergs Stiftelse, and Söderbergs stiftelser. An AMEND Research Award (2023) supported the immunostaining. Sequencing was performed by the SNP&SEQ Technology Platform in Uppsala. The facility is part of the National Genomics Infrastructure (NGI), Sweden, and the Science for Life Laboratory. The SNP&SEQ Platform is supported by the Swedish Research Council and the Knut and Alice Wallenberg Foundation. Part of the material was collected with support of U-CAN, through Uppsala Biobank and the Department of Clinical Pathology, Uppsala University Hospital. The computations and data handling were enabled by resources in project sens2019036 provided by the National Academic Infrastructure for Supercomputing in Sweden (NAISS) at UPPMAX, funded by the Swedish Research Council through grant agreement no. 2022-06725. In addition, the EU COST Action CA20122 Harmonization supported networking throughout the project (www.goharmonisation.com).

## Author contribution statement

RTC and JC conceived, designed, and funded the study. AE, LZ, JMF, RTC, and JC prepared and wrote the paper and prepared the figures. AE, LZ, JMF, HKC, and AS performed data collection and analyses. AE, LZ, TÅ, RS, SW, AH, HKC, IL, PS, AM, DG, LA, PH, HW, JAT, GA, FA, BS, MG, SB, AS, RTC, and JC acquired resources, tissues, and data. MG, AS, JC, and RTC provided supervision. All the authors contributed to writing the manuscript.

## Data availability

Access to the sensitive raw data can be requested through https://www.u-can.uu.se/. All additional data can be obtained from the corresponding author upon reasonable request. The data analyses presented in this paper were performed using publicly available software as detailed in the section titled ‘Methods’. Key scripts to reproduce figures and analyses have been deposited in a GitHub repository: (https://github.com/LiangZhang695/2026_ACC_CETO/).

## Ethical approval

The study was conducted in accordance with the Declaration of Helsinki and was approved by appropriate research ethics committees for Uppsala (EPM Dnrs 2023-00163-01, 2025-00257-02, and 2025-06748-02) and Cambridge (REC: 14/EE/1059).

## Human ethics and consent to participate

All living patients provided written informed consent.

## Disclosure

During the preparation of this work, Joakim Crona used ChatGPT (version December 17, 2025) to improve readability and language. After using this tool/service, the authors reviewed and edited the content as needed and take full responsibility for the content of the publication.
