# Supplementary material for: 18F-CETO in adrenocortical carcinoma: first clinical-translational study of an adrenocortical-targeting PET tracer
Source: Endocr Relat Cancer. 2026 Oct 5;33(10):e260314. doi: 10.1530/ERC-26-0314 (PMC13663316; doi:10.1530/ERC-26-0314)
Supplement: Supplementary file 1 [file ERC-26-0314_supplementary_materials.pdf]

**Supplementary materials**

*Methods*

*PET-Imaging protocols*

*Uppsala:* From March 2005 to May 2021, a GE Discovery ST positron emission tomography combined with computed tomography (PET/CT) scanner was used (General Electric Healthcare, Chicago, IL, USA), which produced 47 slices with a 157 mm axial field of view (FOV) and 700 mm trans-axial FOV. After May 2021 PET/CT was performed on a Discovery MI 4 or 5 rings PET/CT scanner (General Electric, Milwaukee, Wisconsin, USA) with 20 or 25 cm, axial FOV, respectively. Static whole-body examination was obtained, from the base of the skull to the upper thighs, 20 minutes after intravenous injection of approximately 800 MBq of [11C]metomidate ($^{11}$C-MTO) and 60 minutes after intravenous injection of 2 MBq/kg body-weight of para-chloro-2-[18F]fluoroethyletomidate ($^{18}$F-CETO). For attenuation correction of the PET images and for anatomical correlation of the PET findings, a non-contrast-enhanced low-radiation-dose CT examination was performed before the PET acquisition. The PET images (GE Discovery ST) were reconstructed using Ordered Subset Expectation Maximization (2 iterations, 16 subsets) and using a 4.29 mm post-processing filter or (GE Discovery MI) using Time-of-Flight Ordered Subset Expectation Maximization (3 iterations, 16 subsets) including resolution recovery and applying a 5 mm post-processing filter. PET-CT data were normalized and corrected for dead time, random coincidences, physical decay, scatter and attenuation based on the low-dose CT. Images were reconstructed into a 128x128 matrix, and reconstruction settings resulted in a spatial resolution of approximately 6.5 mm. PET data were recalculated to produce standardized uptake value (SUV) images by dividing the radioactivity concentration in each pixel (Bq/mL) with the injected activity (Bq) per body-weight (g)

In *Cambridge*, PET/CT imaging with both tracers was performed on a GE Discovery 690 PET/CT system between 2018 and 2022. After 2022, PET/CT was performed on a Discovery MI GEN 2 PET/CT scanner (General Electric, Milwaukee, Wisconsin, USA). Imaging with $^{11}$C-MTO was undertaken 35 minutes after administration of 95–280 MBq. Imaging with $^{18}$F-CETO was undertaken 60 minutes after administration of 120-200 Mbq for a 20 minute bed position. Image reconstruction for both tracers was performed using an ordered-subsets expectation maximisation iterative reconstruction algorithm with 3 iterations and 24 subsets. Images were corrected using CT attenuation correction, scatter correction, time-of-flight, and point-spread function correction (SharpIR; GE HealthCare).

*Tissue and nucleic acid preparation*

Tissue was sectioned by cryostat. For samples acquired from surgical resection, approximately 10x20µm sections were collected for DNA and RNA extraction. Two 6-µm sections were taken before and after the tissue collected for nucleic acid extraction to be used for histopathology analyses. For core needle biopsies, 30x4µm sections were taken for DNA and RNA extraction. In addition, two 4-µm sections, one at the top and one at the bottom, were acquired for histopathology. Tissue sections for histopathology were subjected to H&E staining. RNA was isolated using AllPrep DNA/RNA/miRNA Universal Kit (Qiagen, Hilden, Germany) following the manufacturer's instructions. RNA quantity and quality (RINe) were assessed by TapeStation RNA ScreenTape Analysis (Agilent, Santa Clara, California, USA).

*Total RNA sequencing*

RNA sequencing was performed at Science for Life Laboratory, Uppsala Node (https://snpseq.medsci.uu.se). Sequencing libraries were prepared from 50-300ng total RNA using the Illumina Stranded Total RNA library preparation kit with Ribo-Zero Plus treatment (cat# 20040525/20040529, Illumina Inc.). Unique dual indexes (cat# 20040553/20040554,

Illumina Inc.) were used. The library preparation was performed according to the manufacturers' protocol (# 1000000124514). Paired-end 150bp read length, NovaSeq X Plus system, 10B flowcell and XLEAP-SBS sequencing chemistry.

*RNA-Seq data analysis*

Data were analyzed using the nf-core/rnaseq pipeline v 3.3[1i7], with transcript quantification by Salmon[19]. Differential expression analyses used DeSeq2 (1.46.0)[20]. Adrenal Differentiation Score (ADS)[21] and adrenocortical zonation scores (reticularis, fasciculate and glomerulosa)[22], with individual gene sets shown in Supplementary Figure 4, were calculated for each sample through GSVA (2.0.7) using single sample Gene Set Enrichment Analysis(ssGSEA). The linear correlations between CYP11B1, CYP11B2, ADS and adrenal zonation scores and PET maximum standardized uptake value (SUVmax) were studied by Spearman Correlation Coefficient. Gene expression levels of CYP11B1 and CYP11B2 was log2-transformed (counts+1).

As per the methodology described by Wu et al,[23]: immunohistochemistry was performed on 3-µm sections cut from paraffin blocks using fully automated systems. The CYP11B1 primary antibody clone RAT-87 (MABS502; Merck) was used at a dilution of 1:100 after heat-induced epitope retrieval at pH 9.0 (BOND Epitope Retrieval Solution 2; AR9640; Leica) for 20 min. CYP11B2 primary antibody clone EPR10494 (ab168388; Abcam) was used at a dilution of 1:200 after proteolytic-induced epitope retrieval with proteinase K (BOND Enzyme Pre-Treatment Kit; AR9551; Leica) for 10 minutes. The primary antibody binding to tissue sections was visualized using a BOND Polymer Refine Detection system (DS9800; Leica). A semiquantitative scoring system of staining intensity was used by a single histopathologist (JT). 0 = absent staining, 1 = diffuse weak or patchy up to moderate, 2 = diffuse moderate or patchy strong and 3 = diffuse strong (same as non-lesional cortex).

*Results*

Transcriptomic profiling has revealed two Adrenocortical carcinoma (ACC) subgroups with different prognosis that are characterized by high (ENS@T C1A (26) / The Cancer Genome Atlas (TCGA) steroid-high) (24) and low (ENS@T C1B (26) / TCGA steroid low(17)) expression of adrenocortical genes (10). Due to limited number of untreated primary tumors in the RNA sequencing cohort, no such analyses will be presented herein.

External validation by using the TCGA cohort consisting of therapy-naive primary tumor samples (n=72) (24) showed linear correlation (R=0.39, $p$=0.0008) between CYP11B1 expression and the adrenocortical differentiation score, with a stronger correlation obtained when stratifying cases by molecular subgroups: Steroid-high (R=0.65, $p$<0.0001) and steroid-low (R=0.64, $p$=0.0005).

| | MTO/CETO-high (n=41) | Heterogeneous MTO/CETO (n=8) | MTO/CETO-low (n=8) | P-value |
|---|---|---|---|---|
| Age of patient at diagnosis, mean +/- SD | 55.14 +/-16 | 56.34 +/-16.8 | 56.7 +/-17.8 | 0.95 |
| Males:Females, n | 11:30 | 4:4 | 2:6 | 0.43 |
| Clinical cortisol excess, n (%) | 17 (41) | 5 (63) | 1 (13) | 0.13 |
| Biochemical signs of cortisol excess without clinical signs, n (%) | 6 (15) | 1 (13) | 0 | 0.81 |
| Biochemical or clinical signs of sex steroid excess, n (%) | 6 (15) | 1 (13) | 2 (25) | 0.84 |
| ENS@T stage IV at diagnosis, n (%) | 20 (49) | 6 (75) | 6 (75) | 0.27 |
| Ki67 index, median (IQR) | 22 (10-35.1) | 30 (25-56.75) | 22 (17-31) | 0.11 |
| Weiss score, median (IQR) | 7 (5.5-7) | 8 (6-8) | 7 (7-8) | 0.58 |
| Tumor subtype, n (%) | … | … | … | 0.08 |
| ACC, n (%) | 38 (93) | 8 (100) | 5 (62) | |
| Oncocytic, n (%) | 2 (5) | 0 | 3 (38) | |
| Myxoid, n (%) | 1 (2) | 0 | 0 | |

**Supplementary Table 1: Characteristics of patients included in Overall Survival analysis (Stage III-IV).**

Percentages may not sum to 100 due to rounding.

ACC, adrenocortical Carcinoma; CETO, para-chloro-2-[18F]fluoroethyletomidate; ENS@T, European Network for the Study of Adrenal Tumors; FDG, [$^{18}$F]fluoro-2-deoxy-D-glucose; IQR, Interquartile Range; SD, Standard Deviation.

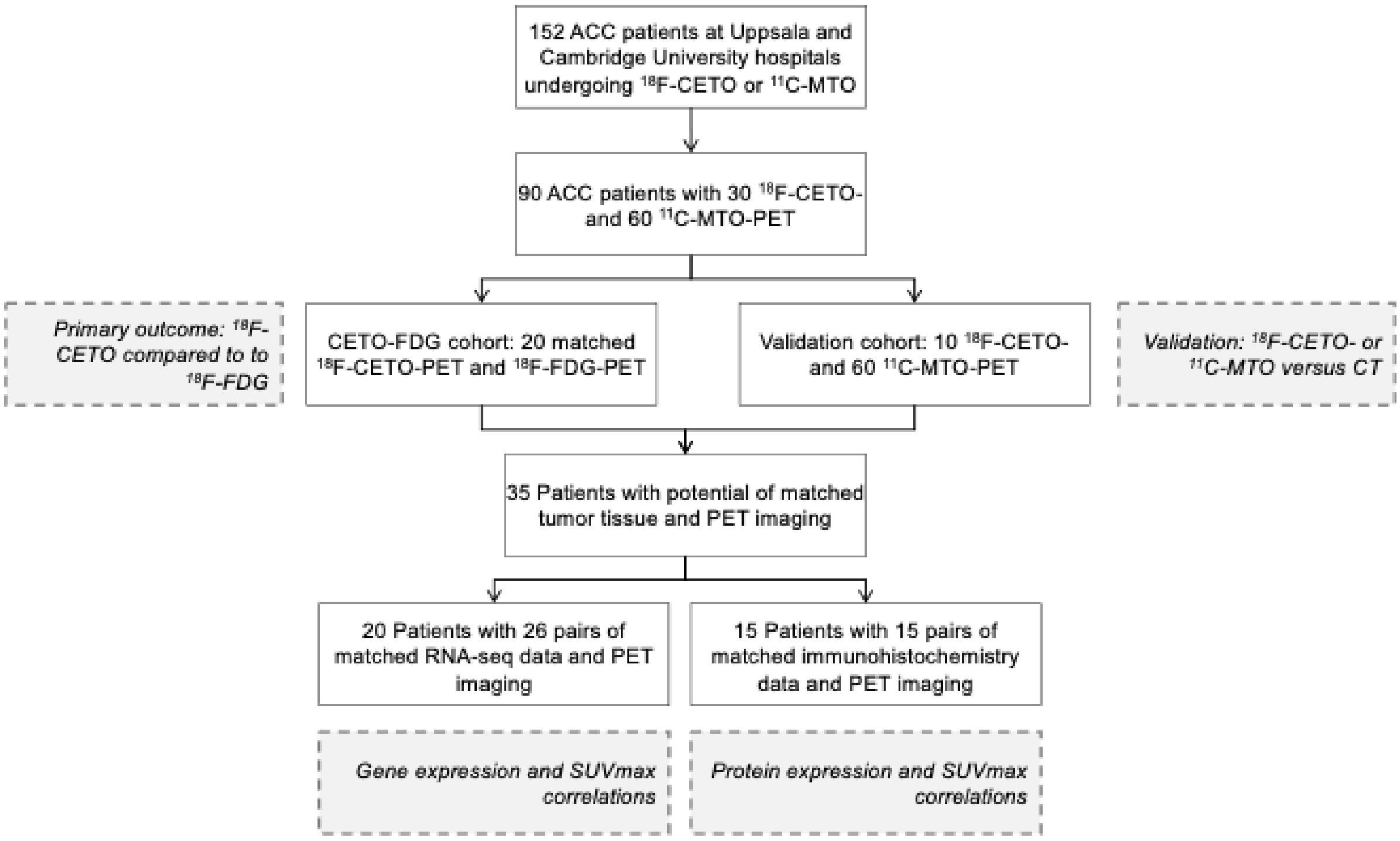


**Supplementary Figure 1. Study flow diagram**

ACC, adrenocortical Carcinoma; 11C-MTO, [11C]metomidate; 18F-CETO, para-chloro-2-[18F]fluoroethyletomidate; 18F-FDG, [$^{18}$F]fluoro-2-deoxy-D-glucose; CT, computed tomography; PET, Positron emission tomography; SUVmax, maximum standardized uptake value;

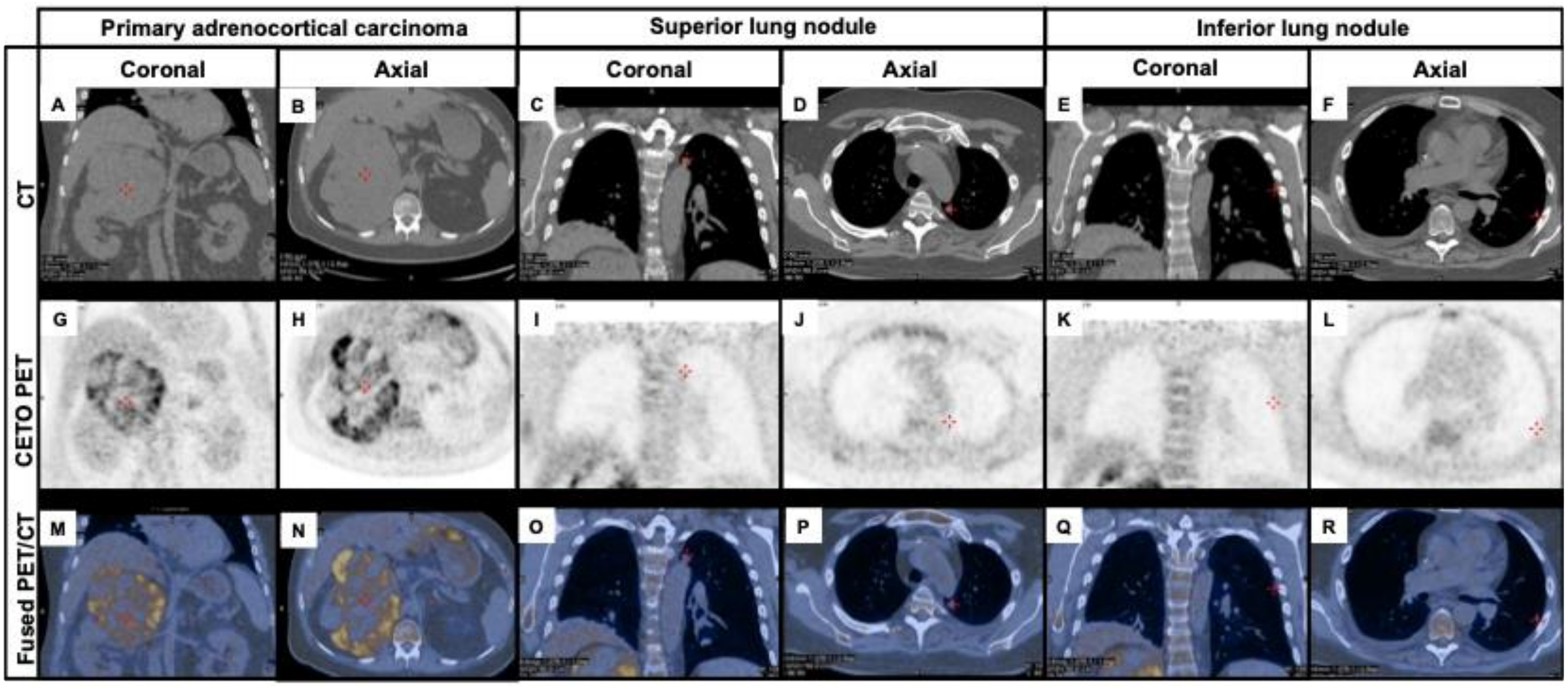


**Supplementary Figures 2A-R**

Representative images from a patient with ‘heterogeneous-high’ [$^{18}$F]fluoroethyletomidate ($^{18}$F-CETO) uptake and aggressive tumoral biology. This patient presented with severe Cushing syndrome and was identified to have a 180 x 130 x 170mm right adrenal mass with an unenhanced radiodensity of 24 Hounsfield units. Two synchronous nodules in the left upper lobe (13mm and 9mm) were also seen. The right adrenal mass shows increased, although somewhat patchy, CETO uptake. No significant tracer uptake is seen within either lung nodule.

A-E

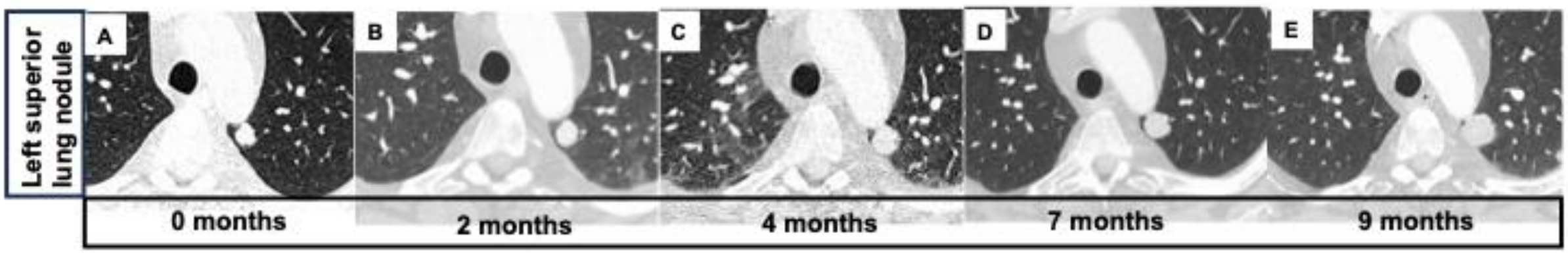


**Supplementary Figure 3A-E**

The lung nodules of the patient in Supplementary Figure 2 were seen to progress rapidly, with the largest increasing from 13mm to 22mm over 9-months, despite early platinum-based chemotherapy

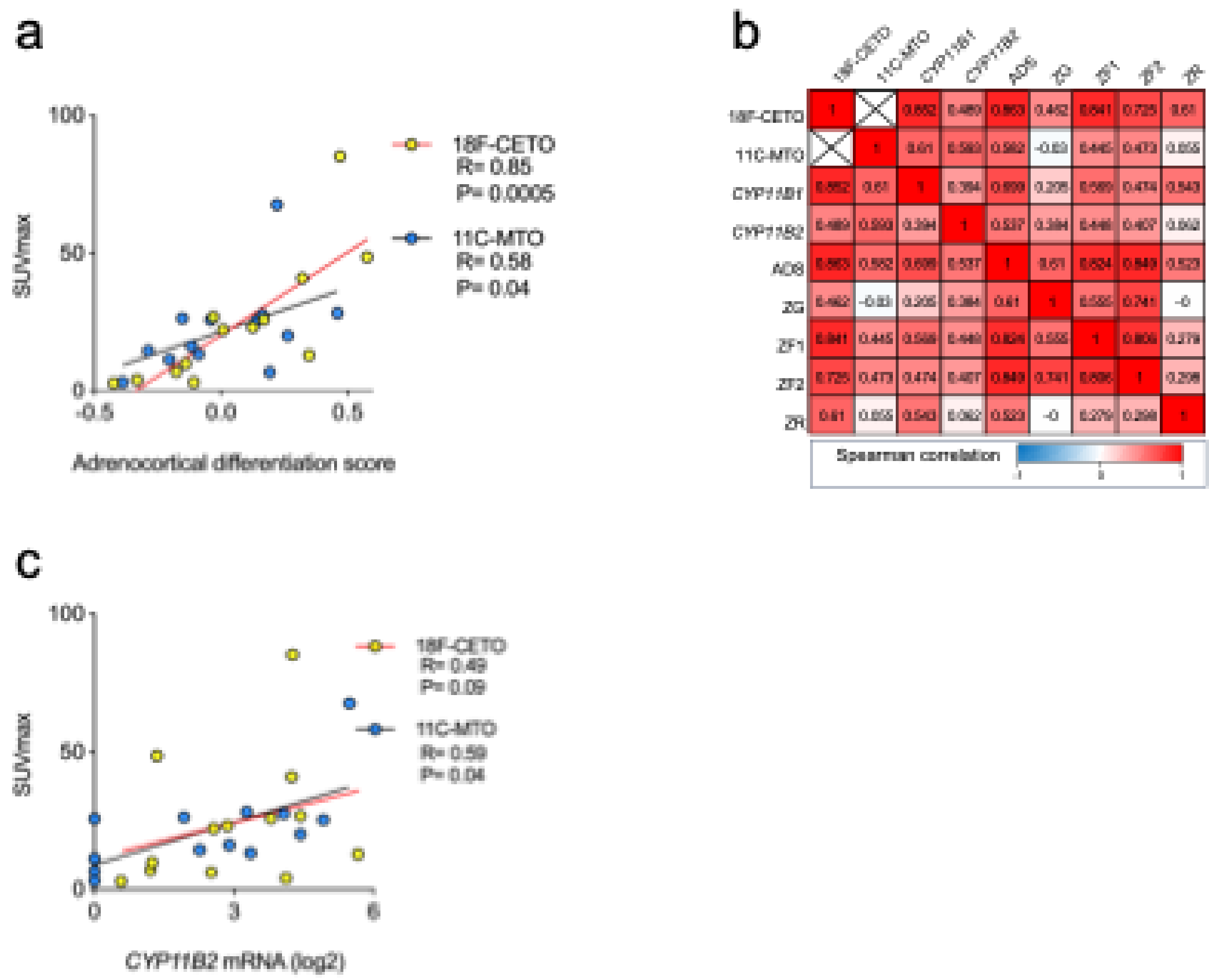


**Supplementary Figures 4A-C**

a) Spearman correlation between ADS and $^{11}$C-MTO (blue) / $^{18}$F-CETO (yellow) SUVmax;

b) correlation heatmap of variables including $^{18}$F-CETO- / $^{11}$C-MTO-PET SUVmax, CYP11B1/B2 gene expression, ADS, and adrenal zonation scores;

c) Spearman correlation between CYP11B2 RNA expression $^{11}$C-MTO-/$^{18}$F-CETO-PET SUVmax.

11C-MTO, [$^{11}$C]metomidate; $^{18}$F-CETO, para-chloro-2 [$^{18}$F ]fluoroethyletomidate; A, Androgen; ADS, Adrenocortical Differentiation Score; PET, Positron emission tomography; SUVmax, maximum standardized uptake value; ZF**,** Zona fasciculata; ZG, Zona glomerulosa; ZR, Zona reticularis.

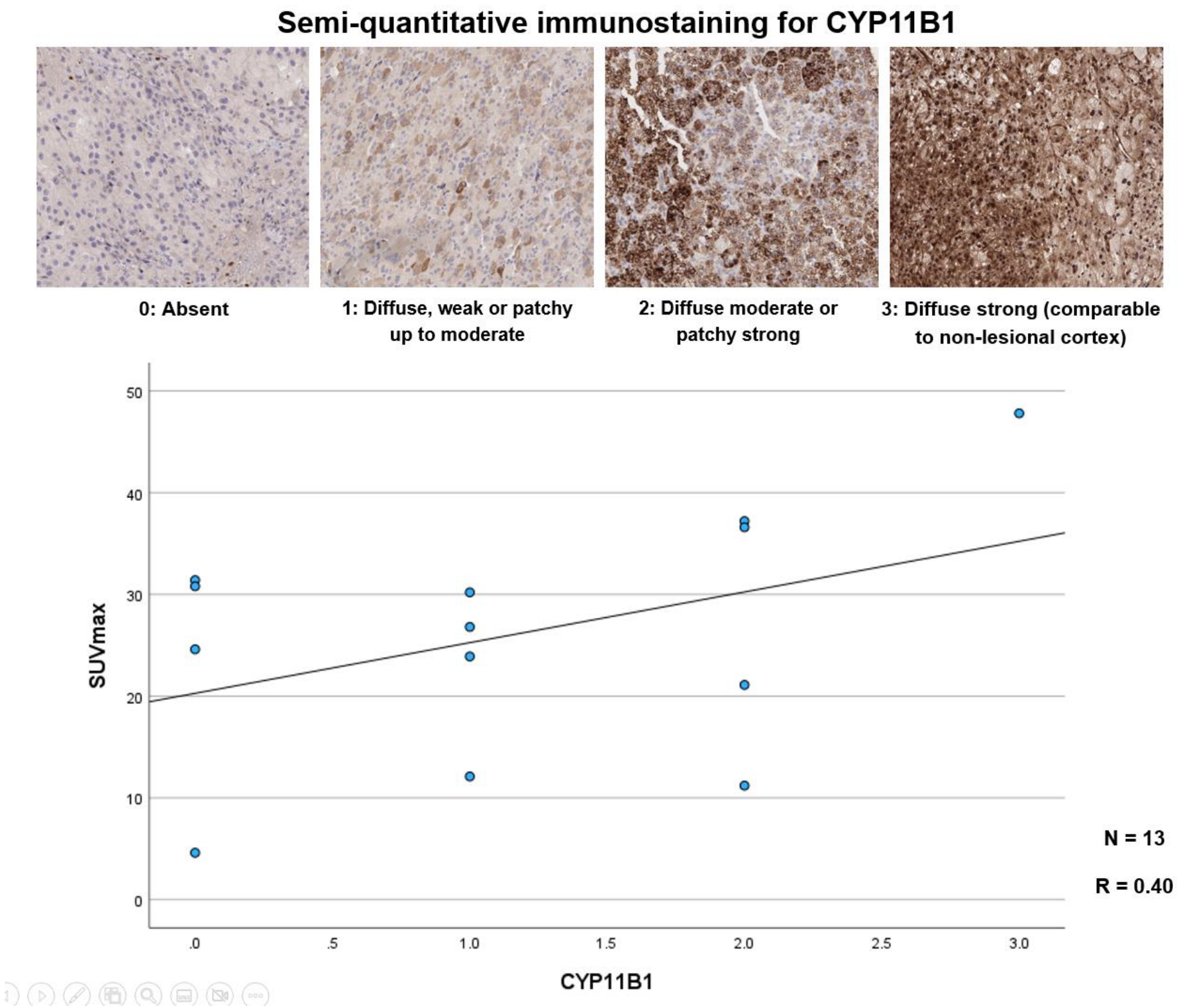


**Supplementary Figure 5**

Examples of semi-quantitative CYP11B1 immunostaining findings at each staining intensity. Spearman correlation between $^{18}$F-CETO SUVmax and semi-quantitative immunostaining score. $^{18}$F-CETO, para-chloro-2 [$^{18}$F ]fluoroethyletomidate; SUVmax, maximum standardized uptake value.

---

**Statements & Declarations**

**Acknowledgements**

The authors acknowledge the important contributions made by the included patients as well as all collaborators, funders, and those maintaining research infrastructure.

**Funding**

The research position of JC is funded by Cancerfonden and LZ was funded by China Scholarship Council: No.202106370081. JM, LA, MG, and RC are supported by the Cambridge NIHR Biomedical Research Centre (NIHR203312). This work was supported by

grants to JC from Cancerfonden, Svenska Läkaresällskapet, Lions Cancerforskningsfond Uppsala, Åke Wibergs Stiftelse and Söderbergs stiftelser. An AMEND Research Award (2023) supported the immunostaining. Sequencing was performed by the SNP&SEQ Technology Platform in Uppsala. The facility is part of the National Genomics Infrastructure (NGI) Sweden and Science for Life Laboratory. The SNP&SEQ Platform is supported by the Swedish Research Council and the Knut and Alice Wallenberg Foundation. Part of the material was collected with support of U-CAN, through Uppsala Biobank and the Department of Clinical Pathology, Uppsala University Hospital. The computations and data handling were enabled by resources in project sens2019036 provided by the National Academic Infrastructure for Supercomputing in Sweden (NAISS) at UPPMAX, funded by the Swedish Research Council through grant agreement no. 2022-06725. In addition, the EU COST Action CA20122 Harmonization supported networking throughout the project (www.goharmonisation.com)

**Disclosure**

During the preparation of this work Joakim Crona used ChatGPT (version Dec 17th 2025) to improve readability and language. After using this tool/service, the authors reviewed and edited the content as needed and take full responsibility for the content of the publication.

**Data availability statement**

Access to the sensitive raw data can be requested through https://www.u-can.uu.se/. All additional data can be obtained from the corresponding author upon reasonable request. The data analyses presented in this paper were performed using publicly available software as detailed in the Methods. Key scripts to reproduce figures and analyses have been deposited in a GitHub repository: (https://github.com/LiangZhang695/2026_ACC_CETO/).

**Author Contributions**

RC and JC conceived, designed and funded the study. AE, LZ, JMF, RC and JC prepared and wrote the paper and prepared the figures. AE, LZ, JMF, HC and AS performed data collection and analyses. AE, LZ, TÅ, RS, SW, AH, HC, IL, PS, AM, DG, LA, PH, HLW, JAT, GA, FA, BS, MG, SB, AS, RC, JC acquired resources, tissues and data. MG, AS, JC and RC provided supervision. All the authors contributed to writing the manuscript.

**Conflicts of interest**

All authors have declared no conflicts of interest
